# Evolution of European light-duty vehicle CO_2_ emissions based on recent certification datasets

**DOI:** 10.1016/j.trd.2022.103287

**Published:** 2022-06

**Authors:** A. Chatzipanagi, J. Pavlovic, M.A. Ktistakis, D. Komnos, G. Fontaras

**Affiliations:** European Commission, Joint Research Centre (JRC), Ispra 21027, Italy

**Keywords:** WLTP, NEDC, CO_2_ emissions, Vehicle emissions, Fuel consumption

## Abstract

A new vehicle testing procedure (WLTP - Worldwide Light duty vehicle Test Procedure) was introduced in the European Union (EU) in 2017. In order to examine its actual impact on CO_2_ emissions for different vehicle technologies and categories, this study analysed data from vehicles certified and registered in the EU in 2019 and 2020. It was found that in average, for all vehicles sold in 2020, the increase in CO_2_ emissions due to the intoduction of the WLTP was 21% for passenger cars and 27% for vans. Also that diesel vehicles are impacted more than gasoline ones and that the impact on conventional hybrid vehicles is 27% and plug-in hybrid vehicles between 0% (in 2020) and 11% (in 2019). Models employed revealed that the increase in CO_2_ is mainly due to the higher test masses and more realistic road load coefficients of WLTP that result in higher cycle energy demands. Moreover, results confirmed that the impact of the WLTP's introduction is in line, both in terms of absolute increase and variability, with model-based predictions performed before fleet-wide data were made available.

## 1 Introduction

Road transport represents almost a quarter of Europe's greenhouse gas emissions with light-duty vehicles (LDVs) estimated to be responsible for around 15% of CO_2_ emissions in the EU (Climate Action EC, 2021).

The 2020 EU fleet-wide CO_2_ average emission targets for passenger cars and vans have been set to 95 gCO_2_/km and 147 gCO_2_/km, respectively, based on the previous New European Driving Cycle (NEDC) that was used for type-approval purposes until September 2017. It is widely known now that the NEDC procedure was far from representing the real-world operation of the vehicles, and therefore fuel consumption and CO_2_ emissions were not realistic (Contag et al., 2017, Anenberg et al., 2017, Mock and German, 2015, Dimaratos et al., 2016, Koszalka et al., 2019). This lead to a gap between the type-approved and real-world values demonstrated in various studies (Dornoff et al., 2020, Fontaras et al., 2017b, Jiménez et al., 2019, Tietge et al., 2019b, Ntziachristos et al., 2014, Zhang et al., 2014).

The solution to this problem came with the introduction of an improved procedure, the so-called Worldwide Light duty vehicle Test Procedure (WLTP), that better represents the real-world operation of vehicles and results in more realistic CO_2_ emissions (Giakoumis and Zachiotis, 2018, Fontaras et al., 2017b, Kuehlwein, 2016). The WLTP is expected to contribute significantly to the reduction of the gap mainly due to its more realistic and less flexible to exploitation test procedure (Tutuianu et al., 2015, Pavlovic et al., 2018, Fontaras et al., 2017a, Pavlovic et al., 2016b, Dings, 2013, Tsokolis et al., 2016). At a very early stage, the correlation between NEDC and WLTP, where only driving cycle was different and road loads applied the same, showed no significant difference (Marotta et al., 2015, Bielaczyc et al., 2015, Andersson et al., 2014). However, this picture was overturned quickly in the following years and an increase equal to 15–25% has been estimated mainly from simulation and/or considering all the technical differences between the two driving cycles and test procedures (Duarte et al., 2016, Ktistakis et al., 2022, Pavlovic et al., 2016a, Pavlovic et al., 2018, Tsiakmakis et al., 2017a, Dornoff et al., 2018).

The type approval of vehicles under the WLTP started in September 2017, but until the end of 2020 some elements of the European Legislation were still based on the NEDC (2020 CO_2_ emission targets, vehicle labelling, national vehicle taxation policies, etc.). When considering all the above, one can understand the importance of investigating the differences in CO_2_ emissions between the two procedures now that the NEDC is phased out and concluding the WLTP uplift factors for different vehicle technologies. Many of the previous studies did not look into the impact of WLTP and the potential differences that could exist between M1 (passenger cars) and N1 (vans). In principle, N1 category vehicles have higher masses than those of M1 category. In this paper, the analysis about the WLTP/NEDC CO_2_ emission ratios is performed separatelly for M1 and N1 categories in order to identify whether the results differ and the reasons behind such differences.

This paper analyses data from the correlation process that was used for the type-approval of all new vehicles in Europe from 2017 to 2020 (Fontaras et al., 2018) and the European Environmental Agency (EEA) CO_2_ monitoring datasets for the calculation of the WLTP/NEDC CO_2_ emission ratios for different vehicle technologies and categories. In addition, the impact of the increase in test mass and the overall cycle energy demand (CED) in WLTP compared to the NEDC on the increase in CO_2_ emissions is analysed. The present work attempts to evaluate the NEDC to WLTP transition also for Hybrid vehicles for which limited previous work in literature has been found (Pavlovic et al., 2017, Liu et al., 2020) or only projections and considerations have been published (Tsiakmakis et al., 2017c). The last part of the study presents a modelling approach performed to quantify the importance of the different vehicle parameters (road loads, mass, engine size, fuel, power, gearshift type, etc.) on the increase of CO_2_ emissions due to the new procedure.

The WLTP/NEDC CO_2_ emission correlation for the year 2020 is of particular importance since that is the last year when both NEDC and WLTP CO_2_ emissions are simulataneously reported for each vehicle registered in the EU and the impact of new WLTP regulation and increase in CO_2_ emissions can be seen on the latest vehicle technologies, the ones that will be sold in the coming years. The main question this study attempts to answer is: How big is the increase in official CO_2_ emissions in EU due to the new test procedure and what is the impact of different vehicle categories, technologies and fuels? This information could be essential for policymakers, researchers and analysts who are currently studying or may study in the future a possible introduction of the WLTP in the countries outside the EU that currently use the NEDC for vehicle certification.

## 2 Methodology

This paper presents the results of an analysis based on two distinct datasets: the European Type-Approval (TA) data and the European Environmental Agency (EEA) CO_2_ monitoring datasets (European Environmental Agency, 2021). The first one is linked to the certification of different vehicle models and model variants, while the second one is related to the vehicles actually sold (registered) in the EU.

### 2.1 Type-approval data

The TA dataset used was collected under the framework of the correlation process through CO_2_MPAS (Fontaras et al., 2018, Tsiakmakis et al., 2017d). CO_2_MPAS is a vehicle CO_2_ emissions and energy consumption simulator developed to facilitate the introduction of the WLTP in the European legislation (Fontaras et al., 2018, Fontaras et al., 2016) for what regards the CO_2_ emissions. From September 2017 until December 2020 data from 3916 different vehicle variants (interpolation families) have been received. Interpolation families (IP families) are composed by the vehicle that exhibits the highest CED over the WLTP (vehicle high or VH) and one with the lowest CED (vehicle low or VL). While VH is mandatory in type-approval of one IP family, the VL is optional and therefore, more results are available for VH than for VL. The TA dataset includes Pure Internal Combustion Engine (Pure ICE) vehicles, Not-Off-Vehicle Charging Hybrid Electric Vehicles (NOVC-HEVs) and Off-Vehicle Charging Hybrid Electric Vehicles (OVC-HEVs).

This paper divides the vehicles into categories M1 and N1. Since this information was not available in the correlation process, the vehicle category for each IP family was identified through either the EEA dataset or the ETAES platform which is a KBA (German Federal Motor Transport Authority) platform gathering vehicle type-approval documents from different EU type-approval authorities (ETAES Platform, 2021).

Some vehicles (IP families) have been type-approved as both M1 and N1 vehicles. These double category vehicles (M1/N1) have been considered in both M1 and N1 analysis. From a total of 3916 IP families received through CO2MPAS, 2759 IP families are M1 category vehicles, 865 IP families are N1 category vehicles, and 292 have been type-approved both as M1 and as N1 (M1/N1). If the 292 duplicated, IP families are accounted for both M1 and N1 categories, 73% of the dataset concerns M1 vehicles and 27 % N1 vehicles (Fig. S1a in the Supplemental Information).

Regarding the vehicle technologies (Fig. S2a in the Supplemental Information), 80% of the investigated M1 category vehicles are Pure ICE and 19% Hybrids (14% NOVC-HEVs and 5% OVC-HEVs). For the N1 category (Fig. S2d in the Supplemental Information), the Pure ICE vehicles correspond to 97.5% (with a predominance of diesel vehicles (91.5%)), and the Hybrids 1.5% (NOVC-HEVs). Other vehicle technologies (1% for both M1 and N1) refers to LPG, NG and ethanol vehicles.

### 2.2 EEA data (EEA 2019 and EEA 2020 datasets)

Eeach year, EU Member States submit to the EEA information related to their new vehicle registrations. In particular, the following details are communicated: manufacturer name, type approval number, type, variant, version, make and commercial name, specific CO_2_ emissions (NEDC and WLTP), masses of the vehicle, wheel base, track width, engine capacity and power, fuel type and mode, eco-innovation savings, and electricity consumption for electrified vehicles (EEA Cars, 2019 and 2020; EEA Vans, 2019 and 2020).

The CO_2_ monitoring data from vehicles (M1 and N1) registered in EU in 2019 and 2020, have been analysed. In total 15.5 million M1 and 1.75 million N1 vehicles were registered in 2019. In 2020, the M1 registered vehicles were 11.6 million and the N1 1.42 million. Registrations of electric vehicles were excluded from the analysis along with registrations that did not provide CO_2_ emissions for both NEDC and WLTP. Hence, the resulting datasets for 2019 contained 14,001,859 M1 and 530,779 N1 vehicles. In 2020, there were 10,504,476 M1 and 1,201,320 N1 vehicles.

As shown in Fig. S1b and Fig. S1c (Supplemental Information), M1 vehicles represented 96% and 90% of the vehicles registered for the years 2019 and 2020, respectively. Fig. S2b (Supplemental Information) shows that in 2019 93% of the M1 registrations were Pure ICE vehicles (61% gasoline and 32% diesel) and only 5% were hybrids. On the other hand, Fig. S2c (Supplemental Information) shows that pure ICE vehicles were reduced in 2020 and represented 81% (53% gasoline and 28% diesel) of the total vehicles registered, while the percentage of hybrid vehicles increased to 17% (12% NOVC-HEV and 5% OVC-HEV).

As seen in Fig. S2e and Fig. S2f (Supplemental Information), the vast majority of the N1 vehicle registrations were Pure ICE diesel vehicles. In 2019 the percentage was 93% and in 2020 97%.

### 2.3 Multiple regression models

Μultiple regression analysis was employed to understand and quantify the importance of different factors (independent variables) to the increase of WLTP/NEDC CO_2_ emissions ratio (dependent variable). As there is no universally accepted definition of variable importance, depending on the purpose, different metrics have been proposed. In this project, the Lindeman, Merenda and Gold metric, introduced by Lindeman et al. (1980), was used. This game-based metric partitions the dependent variable’s variance explained by the model (R- squared coefficient $R^{2}$) into the independent variables’ contributions. This is achieved by averaging the sequential increase in $R^{2}$, when entering each independent variable to the model, over all ordering of independent variables.

Different models were created for VH and VL and for M1 and N1 vehicles. The models take the following forms:

(1) $$\left[WLTP/NEDC\right]CO_{2}emissions=b_{0}+b_{1}f2+b_{2}Mass+b_{3}f1+b_{4}f0+\sum^{k-1}\;{\;}_{i=1}c_{i}OEMgroup+b_{5}Category+b_{6}Fuel+b_{7}Enginepower+b_{8}EngineCapacity+b_{9}Gearbox$$

(2) $$\left[WLTP/NEDC\right]CO_{2}emissions=b_{0}+b_{1}f2+b_{2}Mass+b_{3}f1+b_{4}f0+\sum^{k-1}\;{\;}_{i=1}c_{i}OEMgroup+b_{5}Fuel+b_{6}Enginepower+b_{7}EngineCapacity+b_{8}Gearbox$$

Where Eq (1) is used when M1 and N1 vehicles are examined seperately and Eq (2) when together. *Fuel* is the fuel type (diesel and gasoline), *Gearbox* is the type of gearbox (automatic, manual, CVT), *f0*, *f1* and *f2* are the WLTP/NEDC road load coefficient ratios, *Mass* is the vehicle mass (kg), *Engine power* is the rated engine power (kW), *Engine capacity* is the engine capacity (cm^3^), vehicle manufacturer (*OEM) group* is the pooling group composed of different vehicle manufacturers and k is the number of OEM groups in each case.

## 3 Results and discussion

The results are discussed in three sections. In the first section (3.1), the increase in CO_2_ emissions due to WLTP (WLTP/NEDC ratio) is presented for different vehicle technologies and categories. In the second section (3.2), the results from the regression analysis are shown and the impact of different parameters on the increase in CO_2_ emissions is discussed. Finally, in the last section (3.3), the main factors identified in the previous sections are investigated in more details.

### 3.1 WLTP/NEDC CO_2_ emission ratio

#### 3.1.1 Results grouped by vehicle technologies

**Fig. 1:**
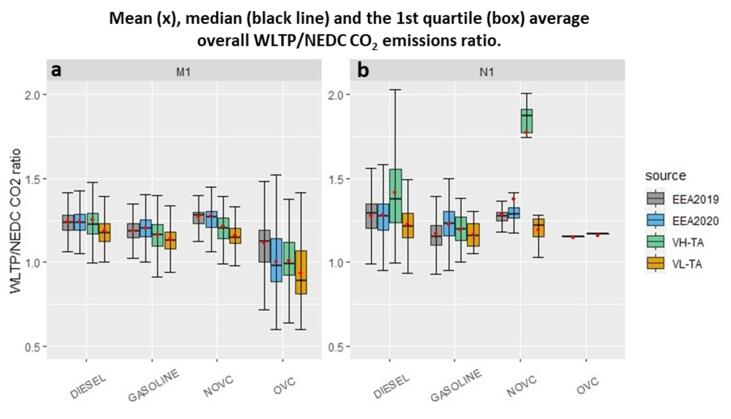
WLTP/NEDC CO_2_ emissions ratio for the different vehicle technologies for **(a)** category M1 and **(b)** category N1 vehicles for VH and VL of the TA dataset and EEA 2019 and 2020 datasets.

The overall mean WLTP/NEDC CO_2_ emissions ratio for M1 and N1 categories separated by different vehicle technologies and extracted from the type-approval (TA VH and TA VL), EEA 2019 and 2020 datasets is shown in Fig. 1a and 1b and Table 1, Table 2. Regarding the pure ICE vehicles, the ones with gasoline technologies are less impacted by the new test procedure relative to the average diesel vehicles. Type-approval results showed that the mean ratio for the gasoline vehicles of category M1 equals to 1.16 for VH (1.19 for N1) and 1.13 for VL (1.16 for N1). The M1 gasoline vehicles registered in EU in 2019 and 2020 had a mean WLTP/NEDC CO_2_ emissions ratio equal to 1.19 and 1.20 (1.17 and 1.23 for N1), respectively.

**Table 1:** WLTP/NEDC CO_2_ ratios (mean and median) from EEA and TA datasets separated by different vehicle technologies and engine sizes for M1 category vehicles.

M1		Type-approval data						EEA 2019			EEA 2020		
Vehicle Technology		# (%)^1^ IP fam VH	Mean VH	Median VH	# (%)^1^ IP fam VL	Mean VL	Median VL	# (%)^1^ vehicles	Mean	Median	# (%)^1^ vehicles	Mean	Median
Pure ICE	**ALL**	**2420**	**1.20**	**1.19**	**2116**	**1.16**	**1.15**	**13,053,344**	**1.21**	**1.20**	**8,516,672**	**1.22**	**1.21**
	**Gasoline ALL**	**1364**	**1.16**	**1.16**	**1142**	**1.13**	**1.13**	**8,546,078**	**1.19**	**1.18**	**5,534,091**	**1.20**	**1.20**
	**Gasoline < 1.4L**	*36%*	1.19	1.19	*39%*	1.15	1.14	*65.5%*	1.19	1.19	*67%*	1.21	1.21
	**Gasoline 1.4-2L**	*46%*	1.16	1.16	*46%*	1.14	1.14	*31.5%*	1.18	1.18	*30%*	1.19	1.19
	**Gasoline > 2L**	*18%*	1.09	1.08	*15%*	1.07	1.06	*3%*	1.14	1.14	*3%*	1.13	1.12
	**Diesel ALL**	**1056**	**1.25**	**1.23**	**974**	**1.18**	**1.17**	**4,507,266**	**1.24**	**1.24**	**2,982,581**	**1.24**	**1.23**
	**Diesel < 1.4L**	*0.5%*	1.18	1.19	*1%*	1.17	1.25	*1%*	1.18	1.18	*1%*	1.20	1.20
	**Diesel 1.4-2L**	*81.5%*	1.24	1.23	*83%*	1.18	1.18	*90%*	1.24	1.24	*93%*	1.24	1.24
	**Diesel > 2L**	*18%*	1.32	1.22	*16%*	1.20	1.15	*9%*	1.21	1.21	*6%*	1.21	1.21
Hybrids	**NOVC-HEVs**	434	1.21	1.20	377	1.16	1.15	575,237	1.27	1.28	1,260,370	1.27	1.27
	**OVC-HEVs**	156	1.01	0.99	144	0.93	0.89	151,762	1.11	1.12	538,514	1.00	0.97
LPG		17	1.15	1.13	14	1.09	1.09	166,624	1.13	1.14	135,526	1.16	1.16
NG		20	1.15	1.14	20	1.09	1.08	47,562	1.17	1.16	53,388	1.15	1.16
ETHANOL		1	1.22	1.22	1	1.21	1.21	7,330	1.06	1.07	6	1.01	1.01
ALL		**3048**	**1.19**	**1.19**	**2672**	**1.14**	**1.14**	**14,001,859**	**1.21**	**1.20**	**10,504,476**	**1.21**	**1.21**

1 # refers to the main categories and % to the sub-categories.

**Table 2:** WLTP/NEDC CO_2_ ratios (mean and median) from EEA and TA datasets separated by different vehicle technologies and engine sizes for N1 category vehicles.

N1		Type-approval data						EEA 2019			EEA 2020		
Vehicle Technology		# (%)^1^ IP fam. VH	Mean VH	Median VH	# (%)^1^ IP fam. VL	Mean VL	Median VL	# (%)^1^ vehicles	Mean	Median	# (%)^1^ vehicles	Mean	Median
Pure ICE vehicles	**ALL**	**1127**	**1.41**	**1.37**	**779**	**1.22**	**1.21**	**527,949**	**1.27**	**1.28**	**1,189,669**	**1.28**	**1.27**
	**Gasoline ALL**	**67**	**1.19**	**1.18**	**52**	**1.16**	**1.15**	**33,623**	**1.17**	**1.15**	**29,487**	**1.23**	**1.23**
	**Gasoline < 1.4L**	*66%*	1.21	1.19	*71%*	1.17	1.16	*83%*	1.18	1.16	*55%*	1.25	1.30
	**Gasoline 1.4-2L**	*25%*	1.19	1.20	*27%*	1.14	1.13	*15%*	1.12	1.10	*3%*	1.16	1.16
	**Gasoline > 2L**	*9%*	1.06	1.03	*2%*	1.15	1.15	*2%*	1.09	1.06	*41%*	1.20	1.21
	**Diesel ALL**	**1060**	**1.42**	**1.38**	**727**	**1.23**	**1.22**	**494,326**	**1.28**	**1.29**	**1,160,182**	**1.28**	**1.28**
	**Diesel < 1.4L**	*2%*	1.18	1.18	*3%*	1.18	1.18	*1%*	1.19	1.18	*2%*	1.19	1.19
	**Diesel 1.4-2L**	*63%*	1.38	1.33	*77%*	1.21	1.20	*78%*	1.27	1.29	*72%*	1.26	1.26
	**Diesel > 2L**	*35%*	1.51	1.49	*20%*	1.30	1.29	*21%*	1.30	1.28	*26%*	1.34	1.34
Hybrid vehicles	**NOVC-HEVs**	19	1.83	1.87	19	1.21	1.24	525	1.29	1.27	3,743	1.37	1.29
	**OVC-HEVs**	0	–	–	0	–	–	176	1.14	1.15	729	1.16	1.17
LPG		7	1.13	1.12	5	1.06	1.07	875	1.09	1.10	2,496	1.13	1.11
NG		4	1.38	1.37	2	1.02	1.02	1,137	1.10	1.08	4,642	1.09	1.07
ETHANOL		0	–	–	0	–	–	117	1.00	1.00	41	1.00	1.00
ALL		**1157**	**1.41**	**1.37**	**805**	**1.22**	**1.21**	**530,779**	**1.27**	**1.28**	**1,201,320**	**1.28**	**1.27**

1 # IP is referring to the main categories and % to the sub-categories.

The mean TA WLTP/NEDC CO_2_ emissions ratios for the diesel VH and VL of the M1 category are 1.25 and 1.18 (1.42 and 1.23 for N1), respectively. Diesel vehicles demonstrated a mean WLTP/NEDC CO_2_ emissions ratio of 1.24 for M1 (1.28 for N1) in both EEA 2019 and 2020 registration datasets. Gasoline and diesel vehicles of category N1 are clearly more impacted than those of category M1.

Ratios for diesel vehicles are significantly higher than gasoline vehicles for both M1 and N1 categories, thus indicating a more significant impact of the transition from NEDC to WLTP for diesel technology. This is also supported by previous studies relying mainly on simulations (Pavlovic et al., 2016a, Fontaras et al., 2018). Some other studies predicted that the WLTP/NEDC CO_2_ emissions ratios will vary from 1.00 (i.e., no change in resulting emissions) to ∼ 1.19 (i.e. 19% higher WLTP CO_2_ emissions than NEDC CO_2_ emissions) depending on the vehicle technology (Mock et al., 2014, Marotta et al., 2015, Blanco-Rodriguez et al., 2016, Pavlovic et al., 2016a, Dornoff, 2019, Dimaratos et al., 2016). These predictions, estimations and calculations are lower than what is presented in this paper. The fact that these studies took place at the beginning or even long before the actual introduction of the WLTP, together with the small samples of vehicles investigated, might explain the underestimation of CO_2_ increase with the new testing protocol. Many of the authors stated that their work should be seen as a preliminary and rather qualitative investigation. Results of the other reports (Chatzipanagi et al., 2020, Tsiakmakis et al., 2017c) are closer to what is presented here.

The WLTP/NEDC CO_2_ emissions ratios for all ICE – as well as for gasoline and diesel vehicles separately – are in general agreement with previous investigations in the literature that use extensive data such as preliminary EEA data and other sources (Tietge et al., 2019a, Tietge et al., 2020, Dornoff et al., 2020).

For the conventional hybrid vehicles (NOVC-HEVs) of category M1 the overall average ratio was found to be 1.21 for VH (1.83 for N1) and 1.16 for VL (1.21 for N1). For the EEA 2019 and 2020 datasets, NOVC-HEV vehicles have an average ratio equal to 1.27 for M1 for both years (1.29 for N1 EEA 2019 and 1.37 for N1 EEA 2020).

The average ratio for plug-in hybrid vehicles (OVC-HEVs) for the TA dataset was 1.01 for M1 VH and 0.93 for M1 VL (no N1 OVC-HEVs in the dataset). Concerning the EEA datasets, the mean WLTP/NEDC CO_2_ ratio for OVC-HEV M1 vehicles decreased from 1.11 (1.14 for N1) in 2019 to 1.00 (1.16 for N1) in 2020. Also for hybrids, N1 vehicles exhibit higher average WLTP/NEDC CO_2_ ratios relative to M1 vehicles.

Ratios for NOVC-HEVs are significantly higher than OVC-HEVs for both M1 and N1 categories, thus indicating a more significant impact of the transition to WLTP for NOVC-HEVs. Previous work (Pavlovic et al., 2017) on the impact of WLTP on the CO_2_ emissions from OVC-HEVs outlined that the new testing procedure might increase or decrease in CO_2_ emissions depending on the characteristics of the vehicle (battery capacity and electric range).

#### 3.1.2 Passenger cars - M1 category

Table 1 presents the WLTP/NEDC CO_2_ ratios based on the engine size for M1 category vehicles. A more detailed table, with smaller engine size bins can be found in the Supplemental Information (Table S1). The overall mean WLTP/NEDC CO_2_ ratio for all pure ICE M1 vehicles of the TA dataset is 1.20 for VH and 1.16 for VL. The ratios for EEA 2019 and 2020 are 1.21 and 1.22, respectively.

When performing the breakdown by the fuel type, it is found that for the gasoline M1 vehicles the ratio is lower than the average (1.16 for VH, 1.13 for VL, 1.19 for EEA2019 and 1.20 for EEA2020). This value differs from predictions in previous studies and expected ratios were generally lower than the ones calculated here (Tsiakmakis et al., 2017b, Mock et al., 2014, Marotta et al., 2015).

The majority of 2019 and 2020 type-approved gasoline vehicles come with an engine capacity that ranges from 1.4 to 2.0L (46%) followed by those of < 1.4L (36%), while the most vehicles sold in 2019 and 2020 were with the engine size < 1.4L (65.5%). As seen from Table 1, the mean WLTP/NEDC CO_2_ ratio decreases with the increase in engine capacity for both TA and EEA datasets. This indicates that the impact of the new testing procedure is more pronounced to small engine size passenger cars. Such a trend is in line with some previous reports (Tsiakmakis et al., 2017b, Mock et al., 2014).

For TA diesel vehicles, the mean ratio for VH and VL is 1.25 and 1.18, respectively (1.24 for EEA2019 and 2020). Also, for this fuel type, the most type-approved and registered vehicles have an engine size between 1.4 and 2.0L. Unlike for the case of gasoline vehicles, for type-approved diesel vehicles, the increase in the engine size increases the mean WLTP/NEDC CO_2_ ratio. This trend is not very clear when analysing the EEA data where the mean ratio increases for the vehicles with engine sizes 1.4–2.0L, but then again decreases for vehicles belonging to > 2.0L class. Some previous studies estimated a drop in the WLTP/NEDC ratio for diesel vehicles with the increase in the engine size, but again these studies were based on simulations and were performed before WLTP entered the regulation (Tsiakmakis et al., 2017b, Mock et al., 2014).

In the Hybrid vehicle category, the TA NOVC-HEVs exhibited a mean WLTP/NEDC CO_2_ ratio of 1.21 for VH and 1.16 for VL, much lower than what has been calculated for vehicles registered in EU in 2019 and 2020 (mean ratio equal to 1.27), indicating that the average NOVC hybrid vehicles entering the EU market have configurations with a WLTP/NEDC CO_2_ ratio higher than that of the average type-approved VH configuration. In addition, gasoline NOVC-HEVs exhibit a higher mean WLTP/NEDC CO_2_ ratio than the pure ICE gasoline vehicles for both VH and VL. On the contrary, diesel NOVC-HEVs have a lower mean WLTP/NEDC CO_2_ emissions ratio than the pure ICE gasoline vehicles (both VH and VL).

For OVC-HEVs, the TA ratio for VH is 1.01 and for VL 0.93, in line with EEA 2020 OVC-HEV values (1.00). Therefore, results imply that OVCs are the least affected by the shift to the WLTP protocol. For the calculation of the above ratios, the weighted-combined values have been taken into consideration, which combine the charge-depleting (electric energy is retrieved from the propulsion battery) and charge-sustaining (electric storage is on a minimum balance level and the combustion engine provides the energy for driving) modes (Eder et al., 2014).

The sample is completed with 17 type-approved IP families using LPG and 20 using NG as fuel. Previous simulation studies (Tsiakmakis et al., 2017c) predicted almost the same results for LPG vehicles (1.16), but much higher ratio for NG fueled vehicles (1.36). Also the EEA 2019 and 2020 datasets show similar mean values for LPGs and 1.17 and 1.15 ratio, respectively for NGs. There is just one type-approved IP family using ethanol as fuel for which no solid conclusions can be made due to the minimal number in the TA and EEA datasets.

#### 3.1.3 Light-commercial vehicles – N1 category

As already mentioned, most studies that analysed the impact of WLTP on CO_2_ emissions did not distinguish between the passenger (M1) and light-commercial (N1) vehicles. Hence, Table 2 shows the results of the analysis carried out for the N1 vehicles. A more detailed table, with smaller engine size bins can be found in the Supplemental Information (Table S2). The overall mean WLTP/NEDC CO_2_ ratio for all pure ICE vehicles is 1.41 for VH (vs. 1.20 for M1) and 1.22 for VL (vs 1.16 for M1). The mean ratios for vehicles registered in EEA 2019 and 2020 are 1.27 (vs 1.21 for M1) and 1.28 (vs 1.22 for M1), respectively. These results indicate that manufacturers certify vehicles with much higher CO_2_ emissions and high WLTP/NEDC ratio, but in practice and on average what is sold in the EU are vehicles with configurations closer to that of VL.

When separating vehicles by fuel type, the overall mean TA ratio for gasoline vehicles is 1.19 for VH and 1.16 for VL compared to 1.17 and 1.23 for vehicles registered in EEA in 2019 and 2020, respectively. The increase in the average ratio in 2020 can be explained by higher sales of gasoline vehicles with engine size above 2.0L that had a much higher increase in the ratio compared to 2019and relative to 1.4–2.0L engines. In general, gasoline vehicles in N1 category are very few (both type-approved and registered) compared to their diesel counterparts and mostly with engine sizes lower than 1.4L. Also for N1 vehicles the mean WLTP/NEDC CO_2_ ratio decreases with the increase in engine capacity for both type-approval data and EEA, except for vehicles registered in 2020, where the mean ratio decreases for vehicles with engine sizes 1.4–2.0L, but then again increases for vehicles belonging to > 2.0L class (the same is observed for M1 category vehicles).

Diesel N1 vehicles are more affected by the introduction of the WLTP. For them the overall mean TA ratio equals to 1.42 for VH (vs. 1.25 for M1) and 1.23 for VL (vs. 1.18 for M1) compared to 1.28 for vehicles registered in EEA in 2019 and 2020 (vs. 1.24 for M1). Most type-approved and registered diesel vehicles have an engine size between 1.4 and 2.0L, followed by vehicles with engines bigger than 2.0L. The mean WLTP/NEDC CO_2_ ratio increases with the engine size, as seen for M1 vehicles. It has been observed that WLTP/NEDC ratios over 1.40, in both M1 and N1 categories, occur for vehicles featuring engines ≥ 1.8L and NEDC masses > 2200 kg. This will be further discussed in the following sections.

Only 19 IP families have been collected in the type-approval process as NOVC N1 hybrid vehicles with an overall mean ratio equal to 1.83 for VH and 1.21 for VL. The NOVC vehicles registered in EU in 2019 and 2020, had a mean WLTP/NEDC CO_2_ ratio equal to 1.29 and 1.37, respectively. The difference in the mean values between type-approval and registered data could be explained by the lack of type-approval for hybrid vehicles certified before 2020 (CO2MPAS/DICE became mandatory for hybrids in 2020). As far as OVC hybrid vehicles are concerned, there has been none in the N1 category of the TA dataset, but from the EEA 2019 and 2020 the mean ratio was found to be 1.14 and 1.16, respectively.

The TA dataset is completed with 7 interpolation families fuelled with LPG and 4 interpolation families with NG. The LPG vehicles have a ratio of 1.13 for VH and 1.06 for VL, whereas EEA 2019 and 2020 datasets demonstrated ratios equal to 1.09 and 1.13, respectively. The NG vehicles have a ratio of 1.38 for VH and 1.02 for VL, while EEA 2019 and 2020 NG vehicles show a ratio equal to 1.10 and 1.09, respectively. Ethanol vehicles in the EEA 2019 and 2020 datasets show no impact at all by the introduction of WLTP (ratio equal to 1).

### 3.2 Analytical results

Table 3 presents the results of the analysis performed through the use of multiple regression models in order to investigate the impact that each parameter has on the increase of CO_2_ emissions from WLTP to NEDC. This analysis was performed only for the TA dataset because very few parameters of interest for this analysis have been reported in the EEA dataset. The R^2^ variance of the model is decomposed into non-negative contributions, and these results are shown in the column named “Importance”. Relative importance is calculated when the contributions from all parameters sum to 100% and is used for comparisons and discussion.

**Table 3:** Variable importance for both categories together (M1 + N1), separate M1 and N1 category of pure ICE (gasoline and diesel) for VH and VL of the TA dataset.

		VH						VL					
		ALL (M1 + N1)		M1		N1		ALL (M1 + N1)		M1		N1	
Variable		Imp/ce	Rel. Imp/ce (%)	Imp/ce	Rel. Imp/ce (%)	Imp/ce	Rel. Imp/ce (%)	Imp/ce	Rel. Imp/ce (%)	Imp/ce	Rel. Imp/ce (%)	Imp/ce	Rel. Imp/ce (%)
***CED split***	**f2**	**23.5**	**28**	**17.2**	**22.7**	**30.6**	**37.1**	**10.8**	**19.4**	**13.6**	**24.2**	**6.5**	**12.3**
	**Mass**	14.5	17.3	**13.7**	**18.2**	**13.7**	**16.6**	**18.6**	**33.5**	**15.3**	**27.2**	**16.4**	**31.3**
	**f1**	13.7	16.3	8.8	11.5	13.8	16.8	0.1	0.3	0	0	1.5	2.9
	**f0**	13	15.5	12	15.8	11.7	14.2	3.5	6.3	0.7	1.3	14	26.6
**OEM group**		**7.4**	**8.8**	**11.6**	**15.4**	**8.3**	**10**	**9.6**	**17.3**	**11.8**	**21**	**9.2**	**17.6**
**Vehicle Category**		4.4	5.3	–	–	–	–	2.5	4.6	–	–	–	–
**Fuel**		4.4	5.2	5.3	7	1.2	1.5	4.6	8.2	5.2	9.3	0.5	1
**Engine Power**		1.6	2	3.4	4.4	0.3	0.4	2.6	4.6	3.7	6.6	0.3	0.6
**Engine Capacity**		0.7	0.8	2.2	2.9	2.5	3.1	0.8	1.4	1.9	3.4	1.5	2.8
**Gearbox**		0.7	0.8	1.6	2.1	0.3	0.3	2.5	4.4	3.9	7	2.4	4.6
**R^2^**		**83.9**	**100**	**75.8**	**100**	**82.4**	**100**	**55.6**	**100**	**56.1**	**100**	**52.3**	**100**

The analysis shows that the increase in the WLTP/NEDC CO_2_ emission ratios calculated from the TA dataset is primarily impacted by the vehicles increase of CED. In particular, the relative importance of CED is calculated to be 77.1% for VH configuration when M1 and N1 categories are examined together (68.2% for M1 and 84.7% for N1 category when analysed separately). Similarly, for VL and when evaluating the M1 and N1 categories together, the relative importance of CED is the highest and calculated to be 59.5% (52.7% for M1 and 73.1% for N1 category when analysed separately).

Since the CED’s contribution is the most significant, further investigation was conducted by splitting CED into its main components: the Road Load coefficients (f0, f1, f2) and the vehicle mass. As it can be seen from the table, for VH (regardless of vehicle category), the most significant contributor to the CED’s importance is the increase in coefficient f2 (28%) which is related to the aerodynamic drag and the fact that in WLTP, for VH configuration, the vehicle with the worst aerodynamics has to be tested compared to the best vehicle in the family tested in the NEDC. For VL, the highest contribution comes from the vehicle mass (19%), that in WLTP is higher compared to the mass used in NEDC. When separated by vehicle category, the f2 coefficient plays an even more significant role for N1 VH vehicles (37%) and the vehicle mass for N1 VL vehicles (31.3%). For both vehicle categories, a significant contribution also comes from f0 coefficient (27% importance for N1 VL), which is related to the rolling resistance.

Aside from CED, another important parameter responsible for the increase in WLTP/NEDC CO_2_ ratio, as identified through the model, is the OEM group. The OEM group relative importance is equal to 8.8% for VH and 17.3% for VL when evaluating M1 and N1 vehicles together. The relative importance of this variable is between 10.0% (N1) and 15.4% (M1) for VH and 17.6% (N1) and 21% (M1) for VL. The OEM groups' importance is more significant for M1 than N1 vehicles.

The vehicle category, i.e. M1 or N1, contributes with only 5.3% for VH and 4.6% for VL. Similar relative importance to the increase in CO_2_ emission ratios (relative importance 5.2% for M1 and N1 together) has the fuel variable. For M1 category vehicles, the fuel contributes 7.0% for VH and 9.3% for VL, whereas for N1 vehicles, relative importances are much lower, 1.5% and 1.0% respectively. The engine power, engine capacity and gearbox do not seem to contribute significantly to the CO_2_ emission ratios increase when passing from NEDC to WLTP. None of them exceeded a relative importance of 7.0%.

### 3.3 Impact of the vehicle mass on CO_2_ emissions

The present study identified the increase in the WLTP vehicle mass as one of the major reasons behind the increase in CO_2_ emissions. Previous studies concluded that the higher test masses result in higher CO_2_ emissions (Zacharof et al., 2016, Dimaratos et al., 2016, Tsokolis et al., 2016, Marotta et al., 2015, Pavlovic et al., 2018). In the case of the NEDC, inertia classes are used for the vehicle testing (Mock, 2011), whereas for WLTP mass is significantly higher than the equivalent NEDC one since it takes into account optional equipment fitted to the vehicle and the vehicle payload (15% for M1 and to 28% for N1 category). This different approach will have a big impact on vehicles with a mass above 2200 kg, that in the NEDC will be always tested with the maximum inertia class equal to 2270 kg, whereas in WLTP they will be tested with their actual vehicle mass. This can result in very high differences between the NEDC inertia and the WLTP test mass regardless of the vehicle category, the fuel, and the powertrain. The maximum difference between NEDC inertia and WLTP test mass ranges from ∼ 350 kg for M1 (Table S3 in the Supplemental Information) to ∼ 800 kg for N1 (Table S4, in the Supplemental Information).

**Fig. 2:**
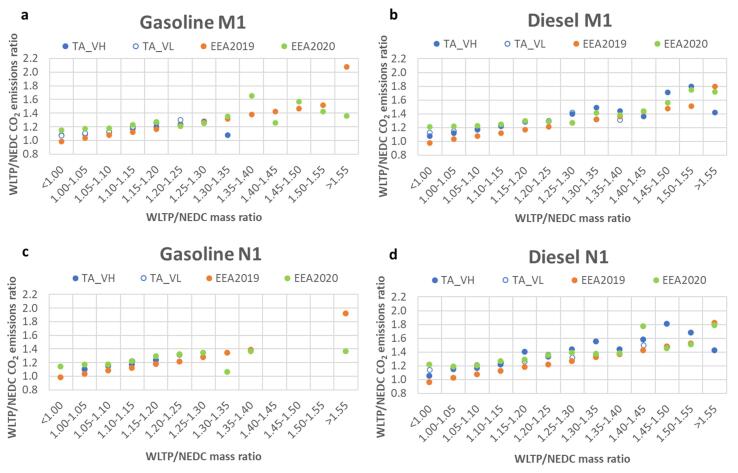
Mean WLTP/NEDC CO_2_ ratio as a function of WLTP/NEDC mass ratio for **(a)** gasoline M1, **(b)** diesel M1, **(c)** gasoline N1 and **(d)** diesel N1 vehicles.

Fig. 2 presents the evolution of the mean WLTP/NEDC CO_2_ ratio for gasoline and diesel vehicles as a function of WLTP/NEDC mass ratio for M1 and N1 category vehicles for the three distinct datasets. The mean WLTP/NEDC CO_2_ ratio is calculated for different WLTP/NEDC mass ratio groups (bins). Mean values for bins that included only one value have been discarded as they do not have a statistical meaning. The first very general remark is that the mean WLTP/NEDC CO_2_ ratio increases with the increase of the WLTP/NEDC mass ratio. For what regards the fuel, the impact of the increase in the test mass is more pronounced for diesel compared to gasoline vehicles.

As far as the M1 gasoline vehicles are concerned, those with WLTP/NEDC mass ratios up to 1.30–1.35 (30–35% increase in the WLTP mass compared to NEDC inertia) result in a similar impact on the WLTP/NEDC CO_2_ ratio. In fact the gasoline type-approved dataset does not contain vehicles with WLTP/NEDC mass ratio above 1.35. The maximum mean WLTP/NEDC CO_2_ ratio for VH is 1.28 (mass ratio group 1.25–1.30), and for VL do not exceed the value of 1.25. In the EEA 2019 dataset, the highest mean WLTP/NEDC CO_2_ ratio was 2.08 (mass ratio above 1.55) and in the EEA 2020 1.65 (mass ratios between 1.35 and 1.40). These results suggest that some vehicles registered in the EU, that have the WLTP mass higher than 40% compared to the NEDC inertia, have a much higher increase in CO_2_ emissions (some even>2 times higher than the NEDC). In general, higher WLTP/NEDC CO_2_ ratios for the same mass bins are found for vehicles registered in 2020 compared to those from 2019, an effect that the change in the testing procedure can not explain, but perhaps in the way how application of the procedure evolved over the years.

For N1 gasoline vehicles, the same increase in the test mass results in slightly higher increase in CO_2_ emissions compared to M1 gasoline vehicles. The maximum WLTP/NEDC CO_2_ ratio for VH is 1.31 (mass ratio group 1.20–1.25) and for VL do not exceed the value of 1.25. In the EEA 2019 dataset the highest mean WLTP/NEDC CO_2_ ratio was 1.92 (mass ratio above 1.55) and in the EEA 2020 1.36 (mass ratios between 1.35 and 1.40).

Regarding the diesel vehicles, the WLTP test mass (for the same NEDC inertia class) is higher for N1 relative to M1 vehicles (Table S3 and S4 in Supplemental Information). For the VH, the highest mean WLTP/NEDC CO_2_ emissions ratio is 1.80 (mass ratio group 1.50–1.55) for M1 category and 1.80 (mass ratio group 1.45–1.50) for N1 vehicles. The VL has its highest CO_2_ emissions ratio equal to 1.42 (for mass ratios 1.25–1.30) and 1.45 (for mass ratios 1.40–1.45) for M1 and N1 categories, respectively. Also for diesel vehicles, for the most bins there are higher WLTP/NEDC CO_2_ ratios for the same mass bins for vehicles registered in 2020 compared to those from 2019 with the maximum average values not exceeding 1.80.

### 3.4 Impact of the cycle energy demand (CED) on CO_2_ emissions

The regression analysis identified the increase in Cycle Energy Demand (CED) of a vehicle due to WLTP as the major factor impacting the increase of CO_2_ emissions. In this section the dependence of CO_2_ emissions on the CED is investigated. The CED of a vehicle is the calculated positive energy at the wheels required by the vehicle to drive the prescribed driving cycle (NEDC, WLTC). Details about CED calculation are described elsewhere (Commission, 2017, Pavlovic et al., 2016a). CED has been calculated from type-approval dataset only (VH and VL), since the EEA datasets don’t contain information about RLs, but only masses.

**Fig. 3:**
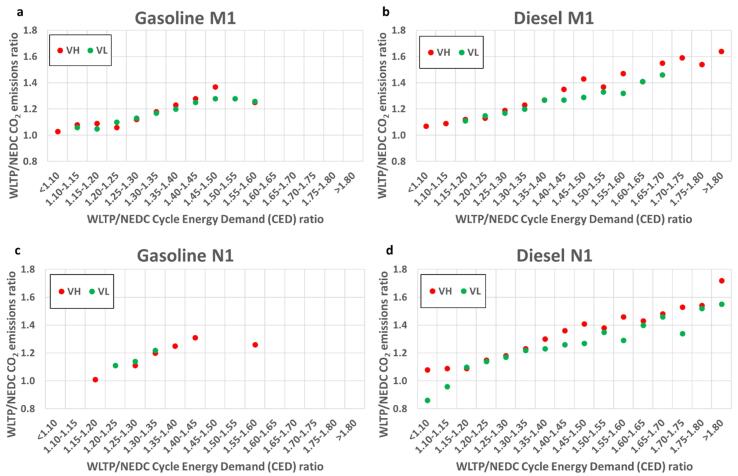
Mean WLTP/NEDC CO_2_ emissions ratio for gasoline and diesel vehicles as a function of WLTP/NEDC CED ratio groups for TA VH and VL of category M1 and N1 vehicles.

Fig. 3 summarises the impact of change (increase) in the CED from the NEDC to the WLTP on the mean WLTP/NEDC CO_2_ ratio for VH and VL, gasoline and diesel, and M1 and N1 category vehicles. As expected, the energy demand of all vehicle categories is increasing with the transition to the WLTP (WLTP/NEDC CED ratio > 1.0, except for some VL N1 diesel configurations). The impact of the increase in the CED is again more significant for diesel compared to the gasoline vehicles, and this can explain the higher WLTP/NEDC CO_2_ ratios for diesel vehicles reported in the previous sections. For the M1 gasoline vehicles, the mean WLTP/NEDC CO_2_ ratios range from 1.03 to 1.37 and for the N1 gasoline vehicles from 1.01 to 1.31 whereas for the M1 and N1 diesel vehicles the ranges are from 1.07 to 1.64 and from 1.08 to 1.72, respectively. Clearly, diesel vehicles have wider ranges and reach higher mean WLTP/NEDC CO_2_ ratios than gasoline vehicles.

Most gasoline vehicles (∼80%) of the M1 category have mean WLTP/NEDC CED ratios between 1.25 and 1.40, signifying an increase between 25% and 40% in the vehicle’s CED due to WLTP. This translates to an increase in CO_2_ emissions between 12% and 23%. The highest mean CO_2_ increase (37%) is found when the CED of a gasoline M1 vehicle increases by 45%-50%.

For the gasoline N1 vehicles, most of them (∼73%) present mean WLTP/NEDC CED ratios between 1.30 and 1.40 with corresponding mean WLTP/NEDC CO_2_ ratios equal to 1.20–1.25. The highest increase in CO_2_ emissions for a gasoline N1 vehicle was found to occur while its CED increased by 40%-45% and it was 31%.

For most M1 diesel vehicles (75%) the mean WLTP/NEDC CED ratios range from 1.25 to 1.40. The corresponding mean WLTP/NEDC CO_2_ emissions ratios range from 1.19 to 1.27 (∼6% more compared to gasoline M1 vehicles). When the CED of a diesel M1 vehicle increases by 65%-70% due to the transition from NEDC to WLTP, the increase in CO_2_ emissions is 46% on average.

For the diesel N1 vehicles, the situation is a bit more complex. For a small number of VL diesel N1 vehicles (<2%) 10% increase in the WLTP’s CED results in the lower WLTP CO_2_ emissions and WLTP/NEDC CO_2_ ratio below 1. Approximately 28% of N1 diesel vehicles are found to have WLTP/NEDC CED ratios from 1.30 to 1.40 (23–30% CO_2_ increase) and 23% over 1.80 (72% CO_2_ increase). A diesel N1 vehicle demonstrates its maximum average increase in CO_2_ emissions (72%) for increases of its CED that are higher than 80% when moving from the NEDC to WLTP.

## 4 Conclusions

In this study official datasets have been used to examine the available evidence about the effect of WLTP on CO_2_ emissions from passenger cars and vans in 2019 and 2020. It was found that regardless of the category, diesel vehicles are impacted more by the transition than gasoline vehicles because of their higher average mass and higher road loads (CED). The average increase in CO_2_ emissions for gasoline vehicles is 20% for both categories (M1 and N1) and for diesel vehicles equal to 24% (M1) and 28% (N1). NOVC hybrid vehicles are impacted by 27% on average and OVC hybrid vehicles between 0% (in 2020) and 11% (in 2019).

In general for gasoline vehicles the WLTP/NEDC CO_2_ ratio decreases with the increase in the engine size. The majority of gasoline vehicles sold in EU have an engine size < 1.4L and in 2020 there was an average increase in CO_2_ emissions equal to 21% (M1) and 25% (N1). On the contrary, for diesel vehicles the WLTP/NEDC CO_2_ ratio increases with the increase in engine size. The majority of diesel vehicles sold in EU have an engine size in the range 1.4–2.0L and in 2020 these vehicles exhibited an average increase in CO_2_ emissions equal to 24% (M1) and 26% (N1).

The regression analysis results suggest that the increase in CO_2_ emissions is mostly (70–80%) driven by the increase in CED and the stricter WLTP's procedure to define the vehicle’s test mass and road load coefficients. According to the model results, the role of OEM groups is not negligible (10–20% depending on the vehicle category and configuration), indicating that different OEMs might have different approaches when it comes to the application of procedures and vehicle testing.

The analysis carried out in the present study confirmed that the introduction of WLTP into EU legislation resulted in a significant average increase in CO_2_ emissions, 21% and 27% in 2020 for cars and vans, respectively. This can be seen as a very important achievement that will significantly reduce the gap between the type-approval and real-world values. The results presented in this paper also identified OVC hybrid vehicles, with sales numbers significantly increasing in EU, as a potential problem in tackling the gap. The WLTP procedure for the majority of these vehicles resulted in CO_2_ emissions lower than the ones measured under the NEDC. Therefore, the regulatory provisions and in particular the utility factors used in type-approval calculations of CO_2_ emissions for these vehicles should be revisited and verified against the representative in-use data.

## CRediT authorship contribution statement

**Anatoli Chatzipanagi:** Conceptualization, Methodology, Writing – original draft, Formal analysis, Investigation. **Jelica Pavlovic:** Conceptualization, Methodology, Writing – original draft, Formal analysis, Investigation, Writing – review & editing, Project administration. **Markos Alexandros Ktistakis:** Formal analysis. **Dimitrios Komnos:** Methodology. **Georgios Fontaras:** Conceptualization, Writing – original draft, Writing – review & editing, Project administration.

## Declaration of Competing Interest

The authors declare that they have no known competing financial interests or personal relationships that could have appeared to influence the work reported in this paper.
